# Noise-Robust Heart Rate Estimation Algorithm from Photoplethysmography Signal with Low Computational Complexity

**DOI:** 10.1155/2019/6283279

**Published:** 2019-05-21

**Authors:** JaeWook Shin, Jaegeol Cho

**Affiliations:** Department of Medical and Mechatronics Engineering, Soonchunhyang University, Asan, Republic of Korea

## Abstract

This paper introduces a noise-robust HR estimation algorithm using wrist-type PPG signals that consist of preprocessing block, motion artifact reduction block, and frequency tracking block. The proposed algorithm has not only robustness for motion noise but also low computational complexity. The proposed algorithm was tested on a data set of 12 subjects and recorded during treadmill exercise in order to verify and compare with other existing algorithms.

## 1. Introduction

Recently, as interest in health increases, there is a growing demand for users to continuously diagnose diseases or to manage disease by measuring biosignals. In order to meet the needs of users, wearable pace measurement devices based on photoplethysmography (PPG) sensors have been commercialized in many companies [1].

The PPG is a sensor that measures changes in blood vessel contraction and expansion using LEDs and photodiodes. It can be used to measure the heart rate and oxygen saturation in a noninvasive manner and is widely used in wearable devices. However, in case of PPG sensor signals in wearable devices, the heart noise estimation error may be caused by motion artifact (MA) due to body movements. Various algorithms have been developed to overcome this problem. Conventional algorithms mainly use PPG sensor signals of different wavelengths to remove motion noise from the PPG sensor signals or effectively remove motion noise using acceleration sensor signals and measure heartbeat [2–8].

However, the existing algorithms use various signals and use complex algorithms. Therefore, the existing algorithms are difficult to use in wearable devices with constraints of price, power, and system size. To overcome these drawbacks, this paper proposes a PPG sensor with low complexity and an algorithm based on a 3-axis acceleration sensor to estimate the heart rate. To evaluate the performance of the proposed algorithm, we compared the performance of the proposed algorithm with that of the existing algorithms.

## 2. Methods

**Figure 1:**
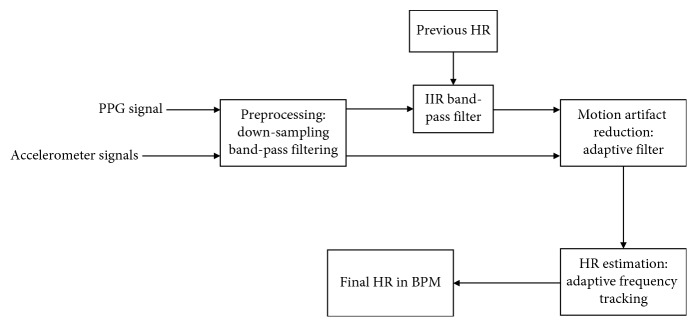
Block diagram of the proposed algorithm.

In this paper, the algorithm consists of three stages in order to estimate the heart rate during exercise in the wearable device based on the PPG sensor. The first stage is preprocessing the input PPG sensor data and 3-axis acceleration data. The second stage is to remove the MA noise from the PPG sensor signal. The last stage is the frequency tracking to estimate the heart rate in the motion-free signal. The flowchart of the proposed algorithm is shown in Figure 1.

### 2.1. Data Set

In this paper, we tested the proposed heart rate estimation algorithm using 12 data sets in the IEEE Signal Processing Cup 2015 database [2]. We compared the heart rate with the output of the ECG signals based on the data set.

### 2.2. Normalized Least Mean Squares Algorithm

The normalized least mean squares (NLMS) algorithm is widely used because it has a simple calculation among various adaptive filters and ease of implementation [9, 10]. We consider data *d*(*n*) derived from an unknown system:

(1) $$\begin{array}{c} d\left(n\right)=u^{T}\left(n\right)w+v\left(n\right), \end{array}$$

where **w** is an unknown system that we expect to estimate, **u** denote the input vector, *v* accounts for the measurement noise, and *n* is the iteration number. Assume that the unknown system order is *M*, **w** and **u** are the *M*-dimensional column vectors. The coefficient $\hat{w}\left(n\right)$ of the adaptive filter is updated using the difference *e*(*n*) between the adaptive filter output signal *y*(*n*) and the desired signal *d*(*n*) for the input signal *u*(*n*) so that the square mean error is minimized. The NLMS algorithm can be expressed as

(2) $$\begin{array}{c} \hat{w}\left(n\right)=\hat{w}\left(n-1\right)+\mu \frac{u\left(n\right)}{u^{T}\left(n\right)u\left(n\right)}e\left(n\right),\\ e\left(n\right)=d\left(n\right)-y\left(n\right)=\;\;\;d\left(n\right)-u^{T}\left(n\right)\hat{w}\left(n-1\right), \end{array}$$

where *μ* is the step size, 0 < *μ* ≤ 1.

### 2.3. Low Computational Complexity MA Reduction Algorithm

**Figure 2:**
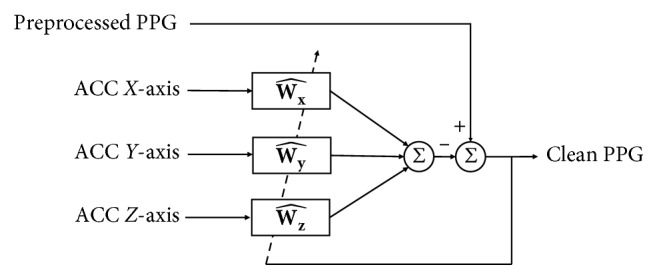
Adaptive filter for motion artifact reduction.

The PPG signal includes noise-free signals and the MA that is generated due to the movement of the body. Because MA is highly correlated with the acceleration sensor signals, a clean PPG Signal can be obtained to remove a signal having a high correlation with the acceleration sensor from the PPG signal. Therefore, the corrupted PPG signals are used as desired signal *d*(*n*) and 3-axis accelerometer signals are used as input signal *u*(*n*) to reduce the MA as shown in Figure 2.

The conventional NLMS algorithm requires 3*M* + 1 multiplication when the order of the adaptive filter is *M*. Despite the small computational complexity of the NLMS, an algorithm with a small computational complexity is required for wearable systems due to price, power, and system size limitations. In order to overcome this drawback, we propose an adaptive noise cancellation algorithm which can have similar performance with low computational complexity as follows:

(3) $$\begin{array}{c} \hat{w_{x}}\left(n\right)=\hat{w_{x}}\left(n-1\right)+\mu \frac{\mathrm{sign}\left(u_{x}\left(n\right)\right)}{\mathrm{sign}\left(u_{x}^{T}\left(n\right)\right)\mathrm{sign}\left(u_{x}\left(n\right)\right)}\text{sign}\left(e\left(n\right)\right), \end{array}$$

(4) $$\begin{array}{c} \hat{w_{y}}\left(n\right)=\hat{w_{y}}\left(n-1\right)+\mu \frac{\mathrm{sign}\left(u_{y}\left(n\right)\right)}{\mathrm{sign}\left(u_{y}^{T}\left(n\right)\right)\mathrm{sign}\left(u_{y}\left(n\right)\right)}\text{sign}\left(e\left(n\right)\right), \end{array}$$

(5) $$\begin{array}{c} \hat{w_{z}}\left(n\right)=\hat{w_{z}}\left(n-1\right)+\mu \frac{\mathrm{sign}\left(u_{z}\left(n\right)\right)}{\mathrm{sign}\left(u_{z}^{T}\left(n\right)\right)\mathrm{sign}\left(u_{z}\left(n\right)\right)}\text{sign}\left(e\left(n\right)\right), \end{array}$$

(6) $$\begin{array}{c} e\left(n\right)=d\left(n\right)-y\left(n\right), \end{array}$$

(7) $$\begin{array}{c} y\left(n\right)=\mathrm{sign}\left(u_{x}^{T}\left(n\right)\right)\hat{w_{x}}\left(n-1\right)+\text{sign}\left(u_{y}^{T}\left(n\right)\right)\hat{w_{y}}\left(n-1\right)+\text{sign}\left(u_{z}^{T}\left(n\right)\right)\hat{w_{z}}\left(n-1\right), \end{array}$$

where sign(·) denotes the sign function and **u**_**x**_,  **u**_**y**_,  *and* **u**_**z**_ denote *x*-axis, *y*-axis, and *z*-axis accelerometer signal, respectively.

Due to use of only the sign of the input signal vector and the error, the proposed algorithm requires only *M* multiplications because the multiplication required in Equations (3)–(5) and (7) can be calculated by adding. Therefore, the algorithm can be implemented with a small amount of computation compared to the existing NLMS algorithm. In particular, calculation time can be further shortened for using a CPU without a floating point unit.

### 2.4. Adaptive Frequency Tracking

We used an oscillator-based adaptive notch filter (OSC-ANF) algorithm [11] to estimate the heart rate using the PPG signal that passed through the MA reduction stage. The OSC-ANF algorithm is based on a second-order IIR band-pass filter and traces the strongest frequency of the signal. The OSC-ANF algorithm operates as follows:

(8) $$\begin{array}{c} x\left(n\right)=\hat{\alpha }\left(n\right)\left(1+\beta \right)x\left(n-1\right)-\beta x\left(n-2\right)+0.5\left(1-\beta \right)\left(\text{sign}\left(e\left(n\right)\right)-\text{sign}\left(e\left(n-2\right)\right)\right),\\ e_{\alpha }\left(n\right)=x\left(n\right)-2\hat{\alpha }\left(n\right)x\left(n-1\right)+x\left(n-2\right),\\ P_{x}\left(n\right)=\left(1-\mu_{\text{a}}\right)P_{x}\left(n-1\right)+\mu_{\text{a}}x^{2}\left(n-1\right),\\ \hat{\alpha }\left(n+1\right)=\hat{\alpha }\left(n\right)+\mu_{\text{a}}\frac{x\left(n-1\right)}{2P_{x}\left(n\right)}e_{\alpha }\left(n\right),\\ \omega \left(n+1\right)=\cos^{-1}\left(\hat{\alpha }\left(n+1\right)\right),\\ HR_{\text{est}}\left(n+1\right)=\omega \left(n+1\right)\times \frac{f_{\text{s}}}{2\pi }\times 60, \end{array}$$

where *ω*(*n*+1) is the estimated frequency, *HR*_est_(*n*+1) is the estimated HR in BPM, *f*_s_ is the sampling rate, *μ*_a_ is the step size, and *β* controls the 3 dB bandwidth of the 2^nd^ order IIR band-pass filter.

### 2.5. Noise-Robust Adaptive Frequency Tracking

To improve the tracking performance of the OSC-ANF algorithm under highly noisy environments, we propose the noise-robust OSC-ANF (NR-OSC-ANF) algorithm that is derived by noise-robust adaptive filter concept [12, 13] as follows:

(9) $$\begin{array}{c} \bar{\alpha }\left(n\right)=\frac{1}{L}\overset{L-1}{\underset{l=0}{\sum }}\hat{\alpha }\left(n-l\right),\\ e_{\alpha }\left(n\right)=x\left(n\right)-2\bar{\alpha }\left(n\right)x\left(n-1\right)+x\left(n-2\right),\\ P_{x}\left(n\right)=\left(1-\mu_{\text{a}}\right)P_{x}\left(n-1\right)+\mu_{\text{a}}x^{2}\left(n-1\right),\\ \hat{\alpha }\left(n+1\right)=\bar{\alpha }\left(n\right)+\mu_{\text{a}}\frac{x\left(n-1\right)}{2P_{x}\left(n\right)}e_{\alpha }\left(n\right). \end{array}$$

By using the average of the past estimated frequencies, the NR-OSC-ANF algorithm makes improved frequency tracking performance in low signal-to-noise ratio (SNR) environments.

In addition, to improve MA reduction performance, we further use IIR band-pass filter, the preprocessed PPG signal by estimated $\hat{\alpha }\left(n+1\right)$, as follows:

(10) $$\begin{array}{c} d_{\text{hr}}\left(n\right)=\hat{\alpha }\left(n\right)\left(1+\beta_{\text{hr}}\right)d\left(n-1\right)-\beta_{\text{hr}}d\left(n-2\right)\;+0.5\left(1-\beta_{\text{hr}}\right)\left(d\left(n\right)-d\left(n-2\right)\right). \end{array}$$

The output of IIR band-pass filter *d*_hr_(*n*) is used as the desired signal for adaptive filter instead of *d*(*n*) in the MA reduction step.

### 2.6. Performance Measurement

To verify the performance of the proposed algorithm, 12 data sets of IEEE Signal Processing Cup 2015 database were used. The data set used provides the reference heart rate measured from the electrocardiogram as well as the PPG sensor signal and the acceleration sensor signal. To compare the performance of the algorithm, we used the two methods that average absolute error and average absolute error percentage as follows:

(11) $$\begin{array}{c} \text{Error}1=\frac{1}{N}\overset{N}{\underset{n=1}{\sum }}\left|HR_{\text{est}}\left(n\right)-HR_{\text{true}}\left(n\right)\right|,\\ \text{Error}2=\frac{1}{N}\overset{N}{\underset{n=1}{\sum }}\frac{\left|HR_{\text{est}}\left(n\right)-HR_{\text{true}}\left(n\right)\right|}{HR_{\text{true}}\left(n\right)}. \end{array}$$

## 3. Results and Discussion

### 3.1. Parameter Settings

**Figure 3:**
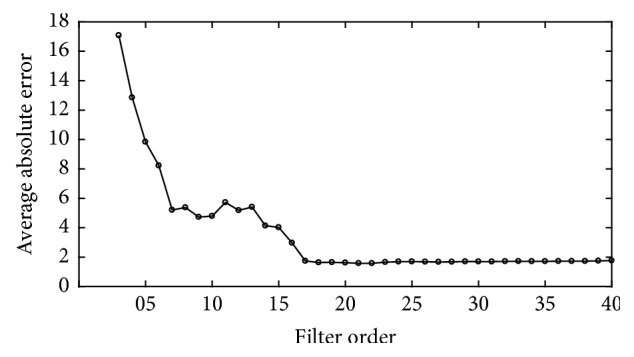
Filter order selection.

In order to reduce the computational complexity, we use down-sampled PPG and accelerometer signal that are resampled 125 Hz to 25 Hz.  Figure 3 shows the average absolute error of the proposed algorithm with various filter tap lengths which used MA reduction step. As can be seen, the proposed algorithm has best performance when the adaptive filter order is 21 (*M* = 21). Parameter setting of the proposed algorithm is summarized in Table 1.

### 3.2. Performance of the Proposed Algorithm

**Figure 4:**
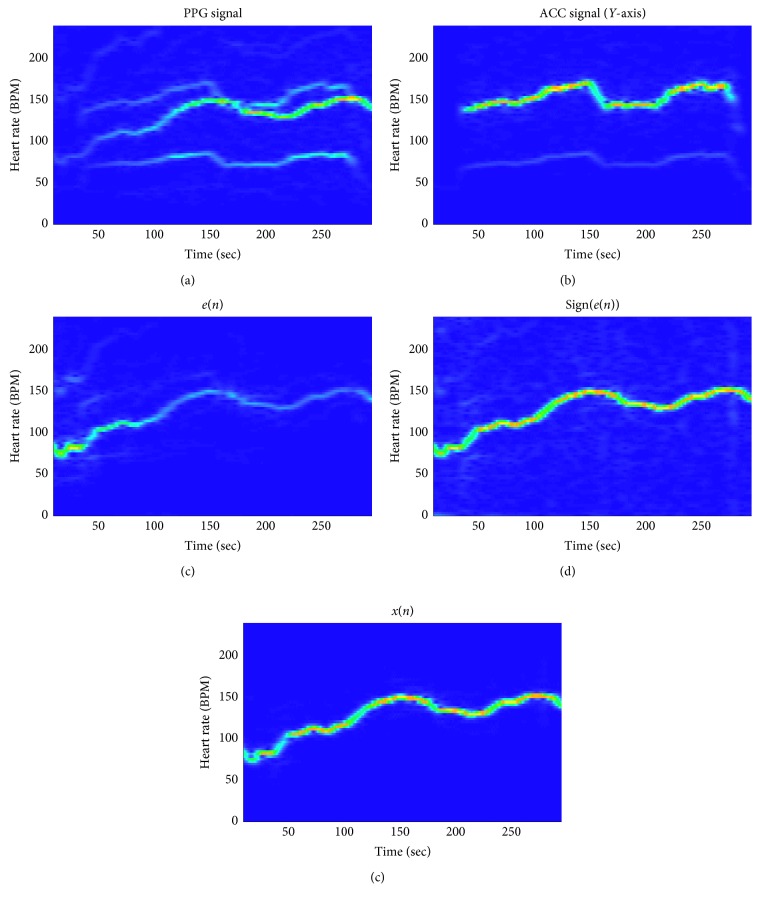
Frequency spectrogram of various signals.

**Figure 5:**
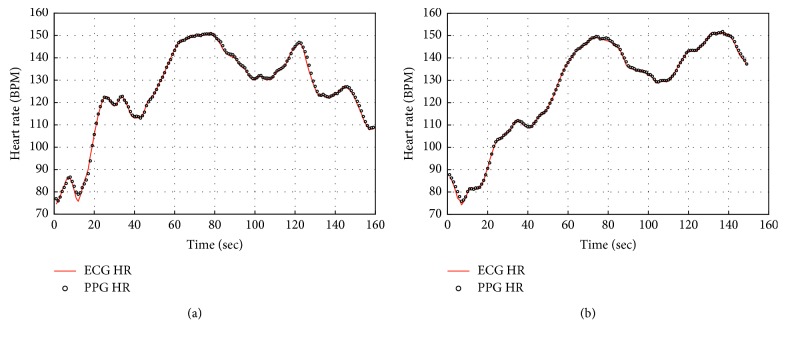
HR estimation results for (a) set 08 and (b) set 09.

In this paper, we verified the performance of the proposed heart rate estimation algorithm using 12 data sets in the IEEE Signal Processing Cup 2015 database. Error1 and Error2 were obtained for each set and compared with other algorithms by comparing the heart rate output through the three-stage algorithm and the ECG signal-based heart rate provided by the data set.  Figure 4 shows that the proposed algorithm can sufficiently remove motion artifacts even with low computational complexity.  Figure 5 is the HR tracking results plot on test data set 08 and set 09 with ECG-based HR. The estimated HR form PPG signal matches with ECG-based HR satisfactorily.

Tables 2 and 3 show that the performances of other existing algorithms and the proposed algorithm do not differ greatly. Although the proposed algorithm does not have best performance compared with other algorithms, it is considered to be worthy of an algorithm for use in a wearable device because of its low computational complexity. The proposed algorithm requires only few multiplication for preprocessing and NR-OSC-ANF.

**Figure 6:**
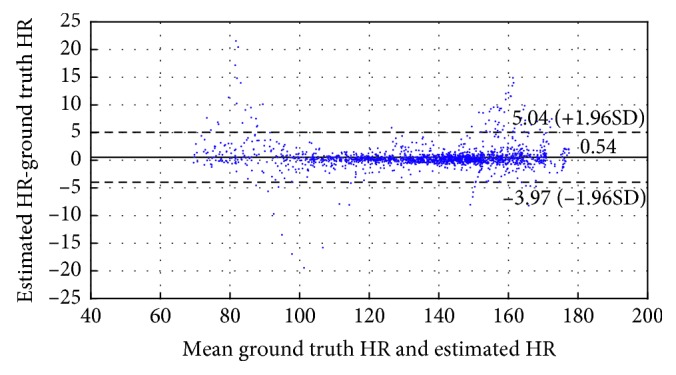
Bland–Altman plot.

**Figure 7:**
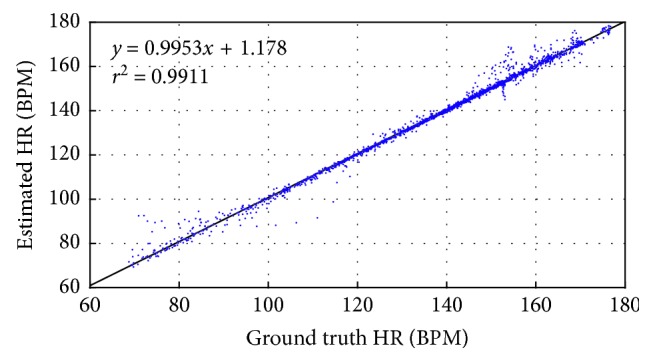
Scatter plot.

Figure 6 shows Bland–Altman plot for the training data set. In this case, the limits of agreement were [−3.97, 5.04] BPM.  Figure 7 indicates the scatter plot between the ground truth HR and estimated HR. The fitted line was *y*=0.9953*x*+1.178, where *x* is the ground truth HR and *y* is the estimated HR.

## 4. Conclusions

This paper presents a noise-robust HR estimation algorithm using PPG signals that have not only robustness for motion noise but also low computational complexity. In order to verify the performance of the proposed heart rate estimation algorithm, we compared with other existing algorithms using the IEEE Signal Processing Cup 2015 database.

## Figures and Tables

**Table 1:** Parameter setting.

Algorithm	Parameters
MA reduction algorithm	*M*=21, *μ*=0.0001
NR-OSC-ANF	*L*=5, *β*=0.95, *μ*_a_=0.025
IIR band-pass filter	*β*_hr_=0.8

**Table 2:** Error1 results of the proposed algorithm and the existing algorithms.

Data set	TROIKA [2]	JOSS [3]	NLMS + OSC-ANFc [7]	Combination of adaptive filters [8]	Proposed algorithm
1	2.29	1.33	1.75	1.34	1.33
2	2.19	1.75	1.94	0.70	1.92
3	2.00	1.47	1.17	0.66	0.83
4	2.15	1.48	1.67	0.70	1.03
5	2.01	0.69	0.95	0.63	0.54
6	2.76	1.32	1.22	0.86	1.44
7	1.67	0.71	0.91	0.66	0.65
8	1.93	0.56	1.17	0.58	0.56
9	1.86	0.49	0.87	0.52	0.43
10	4.70	3.81	2.95	2.46	2.51
11	1.72	0.78	1.15	1.21	0.83
12	2.84	1.04	1.00	0.74	1.79
Av. ± std	2.34 ± 0.79	1.29 ± 0.86	1.40 ± 0.58	0.92 ± 0.52	1.16 ± 0.62

**Table 3:** Error2 results of the proposed algorithm and the existing algorithms.

Data set	TROIKA [2]	NLMS + OSC-ANFc [7]	Combination of adaptive filters [8]	Proposed algorithm
1	1.90	1.59	1.17	1.06
2	1.87	1.99	0.70	2.18
3	1.66	1.02	0.57	0.72
4	1.82	1.51	0.63	0.97
5	1.49	0.75	0.49	0.41
6	2.25	1.05	0.67	1.23
7	1.26	0.72	0.50	0.50
8	1.62	1.04	0.50	0.50
9	1.59	0.76	0.46	0.38
10	2.93	0.93	1.56	1.59
11	1.15	0.79	0.80	0.57
12	1.99	0.79	0.55	1.21

## Data Availability

The data used to support the findings of this study are available from the corresponding author upon request.
